# Beyond parallel programmes: a roadmap for operational integration of family planning and nutrition in low- and middle-income countries

**DOI:** 10.1136/bmjgh-2024-017486

**Published:** 2026-04-13

**Authors:** Marleen Temmerman, Samuel Akombeng Ojong

**Affiliations:** 1 Centre of Excellence in Women and Child Health, Department of Obstetrics and Gynaecology, Aga Khan University Hospital, Nairobi, Nairobi County, Kenya; 2 International Centre for Reproductive Health Belgium, Ghent, Flanders, Belgium; 3 Health, UNICEF, Maputo, Maputo City, Mozambique; 4 Women’s and Reproductive Health Concentration, Johns Hopkins Bloomberg School of Public Health, Baltimore, Maryland, USA

**Keywords:** Delivery of Health Care, Health economics, Health services research, Nutrition, Maternal health

**Summary box**

- Family planning (FP) and nutrition are essential determinants of women’s health, yet implementation in low- and middle-income countries remains largely vertical due to fragmented funding, service delivery and data systems.
- This commentary identifies five actionable entry points for integrating FP and nutrition across the life course and clarifies the need for alignment of policies, financing, workforce roles, supply chains, health management information systems and community platforms, rather than a mere co-location of services.
- We provide practical guidance for embedding this integration into national plans, budgets, indicators and supervision systems to advance Universal Health Coverage and Sustainable Development Goal commitments.

## Introduction: the case for integration

In low- and middle-income countries (LMICs), family planning (FP) and nutrition services are frequently delivered in isolation despite their clear interdependence as determinants of women’s health across the life course.^1 2^ As the 2030 Sustainable Development Goal deadline approaches, and given the indivisible nature of goal 2 (zero hunger), goal 3 (good health and well-being) and goal 5 (gender equality), there is renewed urgency to move beyond parallel programming.^3^

Evidence demonstrates a bi-directional relationship between FP and nutrition. Iron-deficiency anaemia compromises contraceptive efficacy and increases pregnancy risks, while inadequate macro- and micronutrient reserves impair fertility and worsen maternal–fetal outcomes.^4^ Conversely, FP enables healthy birth spacing and nutritional recovery, as unmet FP needs and short interpregnancy intervals drive maternal nutrient depletion and adverse outcomes.^5–7^

This reciprocity is most evident postpartum. The lactational amenorrhoea method promotes exclusive breastfeeding while providing effective contraception and a bridge to longer-acting methods.^5^ WHO’s people-centred Universal Health Coverage model reflected in UNICEF’s Nutrition Strategy similarly endorses life course integration to improve access, outcomes and efficiency.^8^ A decade ago, it was argued that such integration demands a paradigm shift beyond local support alone.^6^

We argue for operational integration of FP and nutrition, shifting from conceptual alignment to deliberate delivery design. Integration entails coordinated policy, financing, service platforms, workforce roles, supply chains, information systems and community engagement to deliver holistic care through a single client pathway, beyond nominal co-location (simply offering both services at the same place or visit). Our case is informed by our professional and lived experiences and leverages existing evidence alongside a *BMJ Global Health* Supplement on FP–nutrition integration, covering analyses of biological reciprocity,^4 5^ reviews of integrated programmes,^9^ a survey of ministry of health officials from 64 countries in Africa, Eastern Mediterranean and South-East Asia on the FP-nutrition integration^10^ and country-level studies.^11–13^ We discuss current service delivery gaps and missed opportunities for integration and propose actionable implementation steps.

## Current gaps and missed opportunities

Despite strong global policy commitments towards integrated service delivery including FP and nutrition programmes, the WHO’s 2024 World Health Assembly report highlighted, *“persistent siloes in delivering specific health services and limitations in the strategic understanding of integrated health service delivery as a mechanism to reach programme-specific goals”*.^8^ These siloes are especially prevalent in LMICs where parallel funding streams, commodity fragility and workforce constraints make vertically delivered services particularly inefficient for women and adolescents, hence our focus.^5 9^ Drawing on existing evidence, including that found in the current Supplement, and leveraging global practice guidelines, this commentary highlights opportunities to operationalise the integrated delivery of FP and nutrition services across the life course continuum (table 1).

**Table 1:** Five integration opportunities across stages of the life course (legend: five evidence-based entry points for integrating family planning and nutrition across the life course continuum, highlighting biologically and programmatically synergistic opportunities to improve health outcomes and reduce missed service contacts)

Continuum stage	Integration opportunity	Rationale
Preconception care	Combine FP with anaemia screening and treatment.	Supports healthy timing of pregnancy and improves fertility, maternal reserves and outcomes.
Antenatal care	Offer micronutrient supplementation alongside FP counselling.	Maximises contact to address nutrition and prepare for postpartum contraceptive needs.
Postpartum period	Support lactational amenorrhoea method-to-modern FP transitions and reinforce maternal nutrition counselling.	Prevents short birth intervals and supports nutritional recovery after childbirth.
Child health	Offer FP counselling/services and nutrition services alongside routine child immunisation visits.	Routine immunisation visits offer repeated contact with postpartum women, enabling efficient delivery of integrated FP and nutrition services without additional visits.
Adolescent health	Integrate sexual and reproductive health education with dietary and anaemia education.	Addresses dual risks of early pregnancy and malnutrition in a high-need demographic.
Community health systems	Equip community health workers to deliver joint FP–nutrition counselling and commodities.	Extends integrated care beyond facilities; reduces missed opportunities for marginalised groups.

FP, family planning.

However, despite these opportunities for integration, many providers lack the skills and support structures to effectively integrate nutrition and FP service delivery.^9 14^ Integrated implementation is inherently complex and requires cross-sectoral coordination, joint training frameworks, aligned commodity systems and a shift in how frontline providers are supervised and supported. Without robust planning and resourcing, integration can inadvertently overburden systems or staff, fostering gaps in service delivery.

Even when offering both FP and nutrition services at the same place or visit, they risk becoming fragmented without strong referral systems, effective communication across cadres and joint accountability mechanisms.^2 8^ Additionally, women and adolescents may also become overwhelmed if they feel pressed to address multiple, non-urgent concerns in a single visit absent adequate support or counselling, given that person-centred integration must strive to fulfil the unique physical and psychological needs of the individual.^6^ In adolescent programmes, these challenges are especially acute. While school-based health platforms are ideal for both FP and nutrition interventions, integration remains rare, as cultural taboos around adolescent sexuality, contraception, menstruation and diet often limit open dialogue on either topic.^10^

Moreover, existing health management information systems rarely track joint FP–nutrition outcomes, making it difficult to assess the impact of integration or scale best practices.^9^ Community health workers (CHWs), who are potentially well placed to support integration, face challenges such as siloed training curricula, inconsistent supervision and commodity stockouts, which prevent them from offering holistic care.^7 14^

Integration can falter when it becomes a top-down exercise that focuses on combining services without centring community needs. Thus, overloading a single visit with multiple tasks can undermine counselling quality, meanwhile adding responsibilities to the work of CHWs without adequate support can lead to increased burden, burnout and stockouts. Approaches that ignore gender and social barriers may also deepen inequities. These limitations should guide programme design so that potential constraints are anticipated and addressed from the outset.

## Towards integrated models: what works and what’s promising

Evidence from implementation research and country experience shows that integrating FP and nutrition is feasible and effective when deliberately designed around existing delivery platforms, workforce roles and community contexts. In Kenya’s Bondo Sub-County, a “one-stop shop” model integrating FP and nutrition through co-trained health workers and harmonised job aids increased postpartum contraceptive uptake and exclusive breastfeeding, reinforced by CHWs at community level.^9 14^ Similarly, Tanzania’s Mara and Kagera regions demonstrated the value of aligning content across services, using lactational amenorrhoea as an entry point to link infant nutrition counselling with modern FP services.^7^

Service integration spans beyond joint service availability. Programmes reporting improvements in service uptake and adherence paired joint delivery with aligned provider training, reliable commodity supply, supportive supervision and strategies that addressed social and gender-related barriers.^9 13^ In settings where early marriages prevail, integration should intentionally support delaying first birth, address anaemia and equip gatekeepers (parents, school leaders, faith and traditional authorities, community champions) to align and reinforce messaging. Engaging male partners and senior family members further addresses stigma and opposition to both FP and dietary change, thus strengthening support for adolescent and postpartum service use.^9^ Effective integration also entails training providers to deliver joint FP and nutrition counselling, using rights-based, client-centred approaches, thus broadening its reach beyond method-specific targets and thus mitigating provider bias.^9 10^

Routine programme implementation supported by UNICEF in Mozambique illustrates how such principles can be operationalised at scale in a workload-sensitive and gender-responsive manner. At community level, CHWs known as *Agentes Polivalentes de Saúde* are supported to provide routine FP and nutrition services, including pills, barrier methods and injectables, alongside micronutrient supplementation, malnutrition screening and referral to primary care facilities. Simultaneously, routine community nutrition programming, included in the *Pacote de Intervenções Nutricionais,* leverages the leadership of women and girls to promote nutritious diets using locally available foods while supporting early identification of malnutrition. Simultaneously, integrated mobile brigades from primary care facilities deliver a package of services in remote communities, combining FP mix, nutrition screening, community-based management of malnutrition and micronutrient supplementation.^15^ Together, these complementary platforms distribute workload across cadres, reinforce women’s agency and allow women and adolescents to access multiple services through coordinated rather than congested points of care.

These experiences underscore that successful FP–nutrition integration is neither ad hoc nor cost-free. Embedding integration within national health plans, supported by explicit budgets, indicators and supervision frameworks, is critical.^7 9 14^ Moreover, strengthening health management information systems to track joint outcomes enables monitoring and course correction, while donor alignment around integrated delivery platforms can reduce fragmentation and inefficiency.^10 11^ Taken together, these examples demonstrate that FP–nutrition integration could be achievable when grounded in existing systems, attentive to workforce realities and supported by coordinated financing, offering a practical pathway to advance health, nutrition and equity goals.

10.1136/bmjgh-2024-017486.supp1 Supplementary data

## Data Availability

All data relevant to the study are included in the article.
