# Supplementary material for: Beyond parallel programmes: a roadmap for operational integration of family planning and nutrition in low- and middle-income countries
Source: BMJ Glob Health. 2026 Apr 13;10(Suppl 1):e017486. doi: 10.1136/bmjgh-2024-017486 (PMC13158656; doi:10.1136/bmjgh-2024-017486)
Supplement: Supplementary data [file bmjgh-10-Suppl_1-s001.pdf]

BMJ Global Health Author Reflexivity Statement

**Study conceptualisation**	
1. How does this study address local research and policy priorities?	This commentary aligns with LMIC research and policy priorities by synthesising evidence and country experiences from Kenya, Tanzania, Burkina Faso, Côte d'Ivoire, Niger, and other LMICs where fragmented FP and nutrition programs constrain women's health outcomes. It responds directly to national goals to strengthen RMNCAH+N integration and operationalize WHO and UNICEF guidance.
2. How were local researchers involved in study design?	LMIC researchers contributed to framing the problem, selecting relevant examples, interpreting implementation constraints, and determining the integration entry points reflected in the commentary.
**Research management**	
3. How has funding been used to support the local research team(s)?	The Gates Foundation grant supporting the Supplement funded the works around this supplement
**Data acquisition and analysis**	
4. How are research staff who conducted data collection acknowledged?	They are authors.
5. How have members of the research partnership been provided with access to study data?	All data used in the commentary come from published or publicly accessible peer-reviewed studies within the Supplement, ensuring shared access for all partners.
6. How were data used to develop analytical skills within the partnership?	We collectively conducted quantitative and qualitative analyses – capacity strengthening was multi-directional.
**Data interpretation**	
7. How have research partners collaborated in interpreting study data?	Interpretation of the evidence, particularly the integration opportunities and implementation gaps, was informed by LMIC partners' contextual insight and expert judgement derived from their country studies.
**Drafting and revising for intellectual content**	
8. How were research partners supported to develop writing skills?	The collaborative development of the Supplement provided opportunities for LMIC researchers to co-author manuscripts, engage in iterative peer review, and participate in joint editorial processes.
9. How will research products be shared to address local needs?	Findings will be disseminated through open-access publication, policy briefs, and presentations through national RMNCAH+N platforms, enabling country teams to apply integration insights in program planning.
**Authorship**	

10. How is the leadership, contribution and ownership of this work by LMIC researchers recognised within the authorship?	LMIC authors contribute substantively to this commentary's conceptualisation and interpretation, and their country-level expertise shapes the operational recommendations.
11. How have early career researchers across the partnership been included within the authorship team?	The commentary author team includes an early-career LMIC researcher (SAO).
12. How has gender balance been addressed within the authorship?	Authorship of this commentary reflects gender diversity
**Training**	
13. How has the project contributed to training of LMIC researchers?	Samuel A. Ojong is an early career researcher who has been able to engage with global research standards.
**Infrastructure**	
14. How has the project contributed to improvements in local infrastructure?	N/A
**Governance**	
15. What safeguarding procedures were used to protect local study participants and researchers?	We followed all research ethics and journal author guidelines
